# Soyer’s Culinary Campaign

**Published:** 1861-11

**Authors:** 


ART. V.—*Soyer's Culinary Campaign. Being Historical Reminiscences of the Late War, with the Plain Art of Cooking for Military and Civil Institutions, the Army, Navy, Public, etc. etc.* By ALEXIS SOYER, author of "The Modern Housewife," "Shilling Cookery for the People," etc. etc. 12mo., pp. 593. London, 1857.

IT is not often, gentle reader, that we place before you a plate of hasty soup; therefore bear with us while we mete out to you a portion of M. Alexis Soyer's palatable dishes. A "Culinary Campaign," under the surveillance of a Frenchman, and one so distinguished in all branches of aristology and the lesser gastronomical orbits, cannot fail to be sufficiently interesting to enable us to keep awake under a summary of the author's Crimean experiences. A résumé, too, is far more agreeable than a perusal of this wonderful effusion, unless one takes delight in conning over the petty sayings of each Sir J——, Lord G——, Count V——, Duke of A——, Duchess of Q——, Prince of H——, etc. etc., all of whom honored M. Soyer with their patronage, and were amply repaid by their protégé's most servile sycophancy. The work is dedicated to Lord Panmure, opens with aristocratic interviews, in all of which Soyer is "Hail-fellow well met," and ends with a familiar tête-à-tête with "my Lord Paulet." Do not fancy that there is an interregnum in which civilians play their modest part; no, even the most celebrated of these are made subservient to the titled few, and are hurried off the scene in as few words as possible. There is, however, one important exception to this last statement; full justice has certainly been rendered Miss Nightingale, a fact which may be accounted for by her ready acquiescence in, and hearty indorsement of, all of our hero's suggestions and culinary improvements. We are always glad to have our own way in this world, and are prone to think well of those who aid us in pursuing our course untrammeled through life's pilgrimage—even in such a sublunary region as the kitchen. Unthwarted regard is by no means a costly gem.

And yet that there was much true metal in M. Soyer's alloy no one can doubt after reading this philanthropic letter, which soon wrought its own fulfillment:—

"TO THE EDITOR OF THE TIMES.

"SIR:—After carefully perusing the letter of your correspondent, dated Scutari, in your impression of Wednesday last, I perceive that, although the kitchen under the superintendence of Miss Nightingale affords so much relief, the system of management at the large one in the Barrack Hospital is far from being perfect. I propose offering my services gratuitously, and proceeding direct to Scutari, at my own personal expense, to regulate that important department, if the government will honor me with their confidence, and grant me the full power of acting according to my knowledge and experience in such matters.

"I have the honor to remain, sir,

"Your obedient servant,

"*Feb.* 2, 1855. ALEXIS SOYER."

This noble offer was at once accepted by the government, and, after having engaged a secretary and two cooks, he set off for the Crimea, fortified by a small model of the field stove—which was also well adapted to hospital use. The incidents of his voyage were many and amusing. These we necessarily pass over, merely stating, for the benefit of any Benedicts who may chance to light upon this article, that especial mention is made of the colossal spout at Constantinople, from whence those Turkish ladies who prove disobedient to their lords are thrown into the Bosphorus—having been previously stitched up in a sack, containing a starving cat and a venomous serpent; rather severe discipline for so hebdomadal a fault!

Upon reaching Scutari, M. Soyer found all the kitchens badly built, and wretchedly ventilated, with the exception of two in the General Hospital. These had been constructed by the Turks, and were superbly fitted up with twenty copper boilers set in white marble, each holding about fifty gallons. But even these were not perfect, as the boilers were greatly in need of tinning. The cooking apparatus of all large establishments should be made of malleable iron, as it is much cheaper, and does not require repairing so constantly as copper. The defects in the boilers were soon remedied, and the kitchens were supplied with charcoal stoves, ovens, store-rooms, dressers, and chopping-blocks, and, under M. Soyer's superintendence, a new culinary *régime* was speedily established.

On the day of his arrival at Scutari, Soyer visited the kitchens after dinner had been served out, and was astonished to find a large copper half full of rich broth, with about three inches of fat upon it—apparently unused. He asked what was done with this, and was told that it was thrown away. We will transcribe his own sequel of this reckless waste: "I took a ladle and removed a large basinful of fat, which, when cold, was better for cooking purposes than the rank butter procured from Constantinople, of from ten to fifteen piastres per pound. The next day I showed the men how to make a most delicious soup with what they had before so foolishly thrown away. This method they were henceforward very glad to adopt. Not less than seventy pounds of beef had been daily boiled in this manner, and without salt."

After bestowing much time and attention upon the Scutari kitchen and rendering cooking a much easier art by papering the walls with culinary receipts and directions, Soyer proceeded to Kululee, where there were three distinct establishments—though under the supervision of but one doctor. The principal kitchen of the Barrack Hospital was in a most wretched condition, being smoky, ill-ventilated, and perfectly dark. The brick work was entirely destroyed, and the men were so blinded by the smoke that they greatly preferred doing dangerous work in the trenches to being subjected to this intolerable nuisance. The cooks were consequently changed every week, which, of course, led to a very bad system of cooking, and was followed by the consumption of 170 per cent. more wood than was necessary. We are told that "the men actually piled small trees, cut into lengths of five or six feet, upon the fires, and when the soup boiled too fast they threw pailfuls of water upon the burning wood, thus filling the place with dust and steam."

Soyer at once had the furnaces put in order, and the skylights repaired, and left one of his assistants in the hospital to instruct the men in the art of camp as well as hospital cooking.

Having remedied the most glaring defects in the Scutari and Kululee hospitals, and after making as many lesser improvements as the short space allotted him would allow, Soyer set sail on the 2d of May, 1855, for the Crimea, accompanied by Miss Nightingale, her friends the Bracebridges, and a number of officers. They met with a most enthusiastic reception on their arrival; throngs of persons poured in to visit Miss Nightingale, and even the common soldiers were all anxious to catch a glimpse of "the good lady of Scutari." Miss Nightingale's name has become so much of a household word that our readers will, perhaps, be glad to see the following description of her *personnel* given in M. Soyer's own language :—

"She is rather high in stature," he writes, "fair in complexion, and slim in person; her hair is brown, and is worn quite plain; her physiognomy is most pleasing; her eyes, of a bluish tint, speak volumes, and are always sparkling with intelligence; her mouth is small and well formed, while her lips act in unison, and make known the impression of her heart—one seems the reflex of the other. Her visage, as regards expression, is very wonderful, and one can almost anticipate by her countenance what she is about to say; alternately with matters of the most grave import, a gentle smile passes radiantly over her countenance, thus proving the evenness of her temper; at other times, when wit or pleasantry prevails, the heroine is lost in the happy, good-natured smile which pervades her face, and you recognize only the charming woman. Her dress is generally of a grayish or black tint; she wears a simple white cap, and often a rough apron. In a word, her whole appearance is religiously simple and unsophisticated."

However great may have been Miss Nightingale's admiration of Soyer's good qualities and his inexhaustible *bonhommie*, we scarcely think her appreciation of the subjoined remark could have been as thorough as our culinary psycopathist could have desired. With many prefatory *ego* encomiums, he mentions, with apparent delight and gratified pride, that, during a visit of General Vivian and his *aide-de-camp* to the kitchens which had been renewed and transformed by his magical presence and advice, the former turned to him and remarked: "Monsieur Soyer, Miss Nightingale's name and your own will be forever associated in the archives of this memorable war."

Laudations of philanthropy are certainly very pleasant and gratifying, but when they come in such a shape as this, Miss Nightingale's "firm nerves" may well "tremble."

The hospital at Balaklava consisted of an immense row of huts, situated on a high mountain, and commanding a grand view of the sea; although extremely cold in winter, and correspondingly warm in summer, the site was a very healthy one, its greatest defect being its inaccessibility, which, however, could not be obviated, as Balaklava is very mountainous, and almost all the residences are built on heights.

The roofless mud kitchens of the hospital not meeting with his approbation, M. Soyer soon improvised a model which was at once accepted, and proved greatly superior to the wretched, neglected hovels which had hitherto constituted the culinary domain. Both professional and extemporaneous cooks were then taken through a course of instruction by their renowned *chef de cuisine*, and thoroughly initiated in the intricacies of camp gastronomy. The army was then suffering from the great inefficiency manifested in preserving meats and bread. When taken out of the tin cases, the meats were always found in a liquid state, unpalatable and totally unfit for use. Soyer's plan was to put up the meat well seasoned, without introducing water or any other liquid, and leave it to cook in its own gravy. When the cases were opened, the meat was perfectly solid, and surrounded by a firm jelly.

Probably the greatest service Soyer rendered during his Crimean campaign was the invention of his bread-biscuit, which was admirably adapted both for field and naval service. This biscuit was somewhat like ordinary bread, but was baked in flat cakes about twelve times the size of a common biscuit. It was made of three parts flour and one of pea meal, and after months' duration was very palatable. The medical board pronounced it highly nutritious and wholesome, and were only too glad to let it take the place of the sour and mouldy article furnished by the contractors at Constantinople.

Since the achievement of her independence, America has had so little experience in the tented field that the ignorance displayed during the present campaign, in everything involving comfort, is not so culpable as it would otherwise be. Complaints pour in from all quarters, and, if these querulous statements are to be credited, our soldiers must be suffering greatly from the absence of suitable clothing and food. Surely, when a man perils his all for the preservation of his country's honor, he is at least entitled to the bare necessaries of life. Even in its prime, the Inquisition could have boasted no more ingenious mode of torture than that which is daily practiced in our midst by giving to the tired, hungry soldier his wretched ration of uncooked, unpalatable food, of which he is compelled to partake to sustain life. A young epicurean tells us that

> "We may live without poetry, music and art;
> We may live without conscience and live without heart;
> We may live without friends, we may live without books,
> But civilized man cannot live without cooks."

Although we do not in the least indorse the statements contained in the first three lines, we are convinced that the last will meet with a universal response. From time immemorial the cook has occupied a prominent place in historic annals, and we doubt not there are still many persons who think with Voltaire that "le cuisinier est un mortel divin." We are all conversant with the cookery of Rome during the period when that proud, hill-girted city won for herself the title of "Mistress of the World," and even in childhood are taught to regard with contempt the gourmand excesses which were hourly committed by her imperial rulers. Eating, with the Romans, was comparatively a simple process before they were allowed glimpses of Asiatic magnificence and luxury. After this, they became almost as effeminate as the Sybarites, those enervated people of whom it is reported that the crumpling of a rose under the side on which they lay occasioned exquisite agony. The Roman Lucullus carried epicurism to such an extreme that he erected several dining halls in his palace, each of which was dedicated to a presiding deity, who, by a supposed simplicity or grandeur of taste, served as a guide to the steward in regulating the expenses of the banquet. For example, a supper in the hall of Apollo generally cost about fifty thousand drachmas, equal to about four thousand six hundred and eighty-seven dollars; those given in the hall of Bacchus were perhaps even more magnificent and costly; while those under the surveillance of Ceres and Pomona were characterized by a much greater simplicity and less lavish profuseness.

To the Medici princesses is accredited the honor of having transplanted Italian cookery to France, which versatile nation adapted itself with wondrous facility to the pleasures of the innovation, and soon became as effeminate as the inhabitants of Sybaris. When the cook of Louis XVIII.

was reprimanded by his majesty's physician for destroying the kingly health with too rich viands, that offended dignitary instantly replied "that it was the office of the cook to supply his majesty with pleasant dishes, and the duty of the physician to enable the king to digest them;" a hint which it would be well for all medical companions of crowned heads to hold in strict remembrance.

English cooks, it is said, can boast of an aristocratic ancestry, dating from William the Conqueror's landing in England. At a banquet given soon after the arrival of the king, in honor of his hardly-won spouse, Matilda of Flanders, he was so delighted with a soup served on that occasion, that he at once sent for his cook, Tezelin, and made him Lord of the Manor of Addington.

We have already digressed too far, as our present object has nothing to do with the luxuries or titled scions of gastronomy; we are anxious only to have culinary camp duties satisfactorily performed, and see our soldiers treated as sentient beings. As yet, our army has been too nomadic in its movements to admit of establishing a parallel between our own culinary department and that existing during the latter part of the Crimean campaign; but should our forces become concentrated, and remain stationary for any length of time, we trust some patriotic Soyer may be found who will do all in his power to obviate the hardships of camp life.

Each regimental physician should be sufficiently versed in cookery to enable him to offer suggestions and remedies in whatever culinary defects he may meet with; he should make it a sacred duty to supervise daily the sick diet, and should endeavor, by care and attention, to render hospital cookery as wholesome and palatable as possible.

We are glad to see that, through the intervention of the Sanitary Commission, the Government has determined to attempt a reformation of the culinary department of the army. This important branch will be undertaken by Mr. J. M. Sanderson, late of the New York Hotel, who will make his first reform in the Onondaga Regiment. Mr. Sanderson is to be chief of a bureau in the Commissary's Department.

The "Culinary Campaign" was not Soyer's only literary effort; he was author of "The Modern Housewife," "Shilling Cookery for the People," and a rather more elaborate production, entitled "Gastronomy of the Ancients."

It is universally accorded that Soyer was a great *artiste*—a word almost entirely monopolized by the votaries of the kitchen—a jolly companion, and a good friend to the sick soldier, inasmuch as he was thoroughly indoctrinated with the saying promulgated by the benign Hippocrates, "What pleases the palate nourishes." His loss was greatly felt and regretted in London, particularly by the *gourmets* who had instituted him as their social "Messer Gaster."

We subjoin a few of Soyer's receipts best adapted to camp cooking, trusting they may be found of use during the present campaign.

*Semi-stewed Mutton and Barley; Soup for* 100 *Men.*—(This receipt was highly approved of by the medical authorities, and was in daily use for more than fifteen months from the date of its introduction.) Put in a convenient sized caldron 130 pints of cold water; 70 lbs. of meat, or about that quantity; 12 lbs. of plain mixed vegetables; 9 lbs. 6 oz. of barley; 1 lb. 7 oz. of salt; 1 lb. 4 oz. of flour; ditto of sugar; 1 lb. of pepper. Put all the ingredients into the pan at once except the flour; set it on the fire, and when beginning to boil, diminish the heat and simmer gently for two hours and a half; take the joints of meat out and simmer well in the orderly's pan; add to the soup flour, mixed with enough water to form a light batter; stir well together with a large spoon; boil another half hour; skim off the fat, and serve the soup and meat separately. The soup should be stirred now and then, to prevent burning or sticking to the bottom of the caldron.

*Beef Tea; Receipt for Six Pints.*—Cut 3 lbs. of beef into pieces the size of walnuts, and chop up the bones; put it into a kettle with ½ lb. of mixed vegetables, such as onions, leeks, celery, turnips, carrots, if they can be obtained; 1 oz. of salt; a little pepper; 1 teaspoonful of sugar, 2 oz. of butter; half a pint of water. Set it on a sharp fire for ten minutes or a quarter of an hour, stirring now and then with a spoon, till it forms a rather thick gravy; then add 7 pints of hot or cold water when boiling; let it simmer gently for an hour; skim off all the fat; strain it through a sieve, and serve.

*Chicken Broth.*—Put in a stew-pan a fowl; 3 pints of water; 2 teaspoonfuls of rice; 1 teaspoonful of salt; an onion, or 2 oz. of mixed vegetables. Boil the whole gently for three-quarters of an hour; if an old fowl, simmer from one hour and a half to two hours, adding 1 pint more water.

*Arrow-root Milk.*—Put into a pan 4 oz. of arrow-root; 3 oz. of sugar; the peel of half a lemon; ¼ teaspoonful of salt; 2½ pints of milk. Set it on the fire; stir round gently; boil for ten minutes, and serve. If no lemons are at hand, a little essence of any kind will do. When short of milk, use half water.

*Soyer's Plain Lemonade.*—Thinly peel the third part of a lemon, which put into a basin with 2 tablespoonfuls of sugar; roll the lemon with your hand to soften it; cut it into 2 pieces, lengthwise; squeeze the juice over the peel; stir round with a spoon for a minute, to form a syrup; pour over a pint of water; mix well, and remove the pips. It is then ready for use.

*Cheap Crimean Lemonade.*—Put into a basin 2 tablespoonfuls of white or brown sugar; half a tablespoonful of lime juice; mix well for 1 minute; add 1 pint of water, and the beverage is ready. A drop of rum will make a good variation.

*Cheap Plain Rice Pudding, for Campaigning,* (in which no eggs or milk are required.)—Put on the fire, in a moderate-sized saucepan, 12 pints of water; when boiling, add to 1 lb. of rice 4 oz. of brown sugar, 1 large teaspoonful of salt, and the rind of a lemon, thinly peeled; boil gently for half an hour, then strain all the water from the rice, keeping it as dry as possible. The rice water is then ready for drinking, either warm or cold. The juice of a lemon may be introduced, which will make it more palatable and refreshing.

*Batter Pudding.*—Break two fresh eggs in a basin; beat them well; add one tablespoonful and a half of flour, which beat up with the eggs with a fork until no lumps remain; add a gill of milk; a teaspoonful of salt; butter a teacup or a bowl; pour in the mixture; then put some water in a stew-pan, enough to immerge the cup or bowl half way in water. Boil twenty minutes, or until the pudding is well set; pass a knife to loosen it; turn out on a plate; pour pounded sugar and a pot of fresh butter over, and serve. A little lemon or cinnamon, or a drop of any essence may be introduced.

*Tea for Eighty Men.*—Put 40 quarts of water to boil; place the rations of tea in a fine net, very loose, or in a large perforated ball; give 1 minute to boil; in 10 minutes it is ready to serve.

*Receipt to Cook Salt Meat for Fifty Men.*—Put 50 lbs. of meat in the boiler; fill with water and let soak all night; next morning wash the meat well; fill with fresh water and boil gently 3 hours, and serve. Skim off the fat, which, when cold, is an excellent substitute for butter.

*Coffee à la Zouave, for a Mess of Ten Men.*—Put 9 pints of water into a canteen saucepan on the fire; when boiling, add 7½ oz. of coffee; mix them well together with a spoon or piece of wood; leave on the fire a few minutes longer, or until just beginning to boil; take it off and pour in 1 pint of cold water; let the whole remain for 10 minutes. The dregs of the coffee will fall to the bottom. Pour it from one vessel to the other, leaving the dregs at the bottom; add the ration of sugar, or two teaspoonfuls to the pint. If any milk is to be had, make two pints of coffee less; add that quantity of milk to your coffee. The former may be boiled previously.

